# Intravitreal Povidone-Iodine Injection and Low-Dose Antibiotic Irrigation for Infectious Endophthalmitis: A Retrospective Case Series

**DOI:** 10.3390/pharmaceutics17080995

**Published:** 2025-07-31

**Authors:** Yumiko Machida, Hiroyuki Nakashizuka, Hajime Onoe, Yorihisa Kitagawa, Naoya Nakagawa, Keisuke Miyata, Misato Yamakawa, Yu Wakatsuki, Koji Tanaka, Ryusaburo Mori, Hiroyuki Shimada

**Affiliations:** 1 Division of Ophthalmology, Department of Visual Sciences, Nihon University School of Medicine, Tokyo 101-8309, Japan; machida.yumiko@nihon-u.ac.jp (Y.M.); onoe.hajime@nihon-u.ac.jp (H.O.); kitagawa.yorihisa@nihon-u.ac.jp (Y.K.); nakagawa.naoya@nihon-u.ac.jp (N.N.); miyata.keisuke@nihon-u.ac.jp (K.M.); yanagishita.misato@nihon-u.ac.jp (M.Y.); wakatsuki.yu@nihon-u.ac.jp (Y.W.); tanaka.koji@nihon-u.ac.jp (K.T.); mori.ryusaburo@nihon-u.ac.jp (R.M.); sshimada@olive.ocn.ne.jp (H.S.); 2 Miyahara Eye Clinic, Saitama 330-0854, Japan

**Keywords:** povidone-iodine, intravitreal injection, endophthalmitis, vitrectomy, vancomycin, ceftazidime, antimicrobial resistance

## Abstract

**Background/Objectives:** Infectious endophthalmitis is a vision-threatening complication of intraocular surgery and intravitreal injections. Standard treatment involves intravitreal antibiotics; however, concerns regarding multidrug resistance and vancomycin-associated hemorrhagic occlusive retinal vasculitis (HORV) highlight the need for alternative antimicrobial strategies. This study aimed to evaluate the clinical efficacy and safety of a protocol combining intravitreal injection of 1.25% povidone-iodine (PI) with intraoperative irrigation using low concentrations of vancomycin and ceftazidime. **Methods:** We retrospectively analyzed 11 eyes from patients diagnosed with postoperative or injection-related endophthalmitis. Six of the eleven cases received an initial intravitreal injection of 1.25% PI, followed by pars plana vitrectomy with irrigation using balanced salt solution PLUS containing vancomycin (20 μg/mL) and ceftazidime (40 μg/mL). A second intravitreal PI injection was administered at the end of surgery in all cases. Additional PI injections were administered postoperatively based on clinical response. Clinical outcomes included best-corrected visual acuity (BCVA), microbial culture results, corneal endothelial cell density, and visual field testing. **Results:** All eyes achieved complete infection resolution without recurrence. The mean BCVA improved significantly from 2.18 logMAR at baseline to 0.296 logMAR at final follow-up (*p* < 0.001). No adverse events were observed on specular microscopy or visual field assessment. The protocol was well tolerated, and repeated PI injections showed no signs of ocular toxicity. **Conclusions:** This combination protocol provides a safe and effective treatment strategy for infectious endophthalmitis. It enables rapid and complete infection resolution while minimizing the risks associated with intravitreal antibiotics. These findings support further investigation of this protocol as a practical and globally accessible alternative to standard intravitreal antimicrobial therapy.

## 1. Introduction

Infectious endophthalmitis is a rare but potentially devastating complication of intraocular surgery and intravitreal injections. Despite its low incidence, it can result in rapid and irreversible vision loss if not promptly and adequately treated [1,2]. The standard therapeutic approach includes prompt intravitreal injection of broad-spectrum antibiotics such as vancomycin and ceftazidime, often in combination with pars plana vitrectomy in moderate to severe cases [3]. However, the increasing emergence of multidrug-resistant pathogens [4,5] and the recognition of severe complications such as hemorrhagic occlusive retinal vasculitis (HORV) associated with vancomycin use have raised serious concerns about the limitations of current antimicrobial strategies [6,7,8,9,10].

Povidone-iodine (PI) is a widely used antiseptic with rapid and broad-spectrum activity against bacteria [11], fungi [12], viruses [13], and biofilms [14]. Notably, it has not been linked to the development of microbial resistance, making it a promising agent in the era of antimicrobial stewardship [15]. These properties position PI as a potentially safer and more sustainable alternative to conventional intravitreal antibiotics, particularly in addressing the growing threat of antimicrobial resistance and avoiding vancomycin-related complications such as HORV. Furthermore, due to its low cost and global availability, PI may be especially valuable in resource-limited settings, including developing countries [16].

Previous investigations by our group demonstrated the safety and efficacy of intraoperative irrigation using 0.025% PI during vitrectomy for infectious endophthalmitis [17], and subsequently, a protocol combining intravitreal injection of 1.25% PI with PI-containing irrigation [18]. These studies confirmed effective infection resolution without significant ocular toxicity, supported by electroretinography (ERG), specular microscopy, and visual field testing. However, in our previous protocol utilizing PI-containing irrigation, a high-dose intravitreal injection of antibiotics (vancomycin 1 mg/0.1 mL and ceftazidime 2 mg/0.1 mL) was still administered at the end of surgery [17,18]. Moreover, because the antimicrobial activity of PI in the irrigation solution is time-limited, it was necessary to manually replace the infusion bottle with freshly prepared PI-containing BSS during the operation. This introduced procedural complexity and raised concerns about potential vancomycin-related complications, such as HORV.

To address these limitations, we developed a modified and simplified approach. In this study, we eliminated the need for high-dose intravitreal antibiotics and instead incorporated low concentrations of vancomycin (20 μg/mL) and ceftazidime (40 μg/mL) into the BSS PLUS irrigation solution. This allows for continuous intraoperative antimicrobial exposure with minimal risk of toxicity. We also retained the use of 1.25% intravitreal PI prior to vitrectomy and additionally administered another PI injection at the end of surgery, as well as a postoperative PI injection when needed, to further enhance infection resolution control. The treatment protocol used in this study is summarized in Supplementary Figure S1, alongside previously reported protocols for comparison. This flowchart highlights the procedural differences, particularly the replacement of high-dose intravitreal antibiotics with low-dose intraoperative irrigation containing vancomycin and ceftazidime, as well as the addition of postoperative PI injection, which was not included in previous protocols. Here, we present the clinical outcomes of this novel strategy.

## 2. Materials and Methods

### 2.1. Study Design and Ethical Approval

This was a retrospective, single-center case series conducted at Nihon University Hospital, Tokyo, Japan. The study included 11 eyes from 11 patients diagnosed with postoperative or injection-related infectious endophthalmitis between 2020 and 2024. The study protocol was approved by the Institutional Review Board of Nihon University Hospital (Approval Code: 20231001; Date of Approval: 16 October 2023) and adhered to the tenets of the Declaration of Helsinki.

The off-label use of 1.25% PI for intravitreal injection was formally reviewed and approved by the hospital’s Pharmaceutical Affairs Committee prior to its clinical application.

### 2.2. Patient Selection and Inclusion Criteria

Eligible patients included those who developed infectious endophthalmitis following intraocular surgery or intravitreal injection. Diagnosis was based on characteristic clinical findings, such as reduced visual acuity, anterior chamber inflammation with or without hypopyon, vitreous haze or opacity, and ocular pain.

Patients with non-infectious uveitis or traumatic ocular injury were excluded.

### 2.3. Treatment Protocol

Upon clinical diagnosis of endophthalmitis, 6 of the 11 patients (Cases 1 and 7–11) received an initial intravitreal injection (IVI) of 0.1 mL of 1.25% PI as an emergency first-line treatment in the outpatient setting, prior to hospital admission. The remaining 5 patients (Cases 2–6) did not receive this preoperative injection due to immediate surgical scheduling or logistical factors.

To prepare the 1.25% PI solution, 1 mL of commercially available 10% PI (ISODINE SOLUTION10%; Mundipharma K.K., Tokyo, Japan) was withdrawn through a 0.22 µm membrane filter and mixed with 7 mL of sterile normal saline. A 0.1 mL aliquot of the resulting 1.25% PI solution was used for each intravitreal injection (see Supplementary Figures S2 and S3). Assuming the vitreous volume to be 5 mL, the resulting intraocular concentration of PI after injection was approximately 0.025%, which is equivalent to the concentration used in our previous study employing 0.025% PI-BSS PLUS as an irrigation solution during vitrectomy for endophthalmitis [17,18].

Subsequently, patients were admitted for surgery and underwent pars plana vitrectomy under local anesthesia using a 25-gauge system (Constellation Vision System, Alcon, Fort Worth, TX, USA). Intraoperative irrigation was performed using balanced salt solution PLUS supplemented with vancomycin (20 μg/mL) and ceftazidime (40 μg/mL). PI was not included in the irrigation solution. At the conclusion of surgery, a second intravitreal injection of 0.1 mL of 1.25% PI was administered in all cases.

Additional postoperative PI injections (same dose and concentration) were performed every 48 h as needed, based on clinical signs of persistent intraocular inflammation.

No postoperative intravitreal antibiotics were used in order to avoid potential complications such as vancomycin-associated hemorrhagic occlusive retinal vasculitis (HORV). Topical antibiotics and corticosteroids were administered in accordance with standard postoperative protocols. In addition, all patients received systemic intravenous meropenem for five days following surgery.

### 2.4. Clinical Evaluation and Outcome Measures

All patients underwent comprehensive ophthalmic evaluations before and after treatment.

The primary outcome measure was best-corrected visual acuity (BCVA), recorded at baseline and at the final follow-up visit. BCVA was measured using decimal visual charts and converted to logMAR units for statistical analysis.

Secondary outcome measures included the following:

- Resolution of intraocular infection, defined as the disappearance of anterior chamber inflammation and vitreous opacity without recurrence;
- Corneal endothelial cell density, assessed using non-contact specular microscopy (SP-3000P, Topcon, Tokyo, Japan) when feasible;
- Visual field assessment using Goldmann perimetry;
- Incidence of adverse events such as retinal toxicity, hypotony, or corneal decompensation.

Due to the retrospective nature of the study, not all secondary parameters were available for every case. Corneal endothelial cell density could not be measured preoperatively in one case due to corneal opacity and inflammation, and it was not performed in an additional three cases. Goldmann perimetry was not conducted in three eyes. Microbial culture results from vitreous and/or aqueous samples were obtained when available. These samples were collected at various time points, including prior to the initial intravitreal PI injection, before vitrectomy, and at the conclusion of surgery. If any of the samples yielded a positive culture result, the identified organism was considered the causative pathogen for that case.

### 2.5. Statistical Analysis

Statistical analysis was performed using Python (version 3.10; Python Software Foundation, Wilmington, DE, USA) with the SciPy library (version 1.10.1).

Best-corrected visual acuity (BCVA) was converted from decimal values to the logarithm of the minimum angle of resolution (logMAR) units. Pre- and postoperative BCVA were compared using a paired *t*-test.

Corneal endothelial cell density was similarly analyzed using a paired *t*-test for the five eyes for which both preoperative and postoperative measurements were available.

A *p*-value of less than 0.05 was considered statistically significant.

All statistical procedures were independently verified by the authors using standard spreadsheet software to confirm accuracy.

### 2.6. Use of Generative AI

Generative AI (ChatGPT, version GPT-4, OpenAI, San Francisco, CA, USA) was used to assist in refining the English grammar, sentence structure, and formatting of the manuscript. All scientific content, including study design, statistical analysis, data interpretation, and conclusions, was planned and verified solely by the authors.

## 3. Results

### 3.1. Visual Acuity Outcomes

All 11 eyes exhibited significant improvement in best-corrected visual acuity (BCVA) following treatment.

Preoperative BCVA ranged from light perception to 0.08 in decimal units (mean logMAR: 2.18), while postoperative BCVA ranged at the final follow-up visit from 0.1 to 1.2 (mean logMAR: 0.296).

The improvement in BCVA was statistically significant (*p* < 0.001, paired *t*-test; *n* = 11).

**Figure 1:**
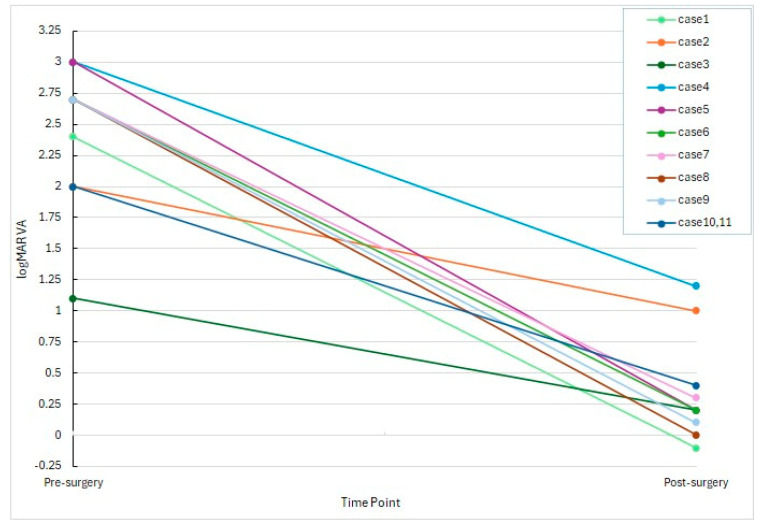
Line chart showing individual changes in logMAR visual acuity before and after treatment in 11 eyes with infectious endophthalmitis. Each line represents one patient. All cases demonstrated visual improvement following the treatment protocol consisting of intravitreal 1.25% PI injection and vitrectomy with low-dose antibiotic irrigation. BCVA values were converted to logMAR units for statistical analysis.

As illustrated in Figure 1, most eyes demonstrated a marked reduction in logMAR values, with 8 out of 11 eyes achieving postoperative decimal visual acuity of 0.4 or better.

Even in cases with poor initial vision (e.g., hand motion or light perception), substantial functional recovery was observed.

### 3.2. Corneal Endothelial Cell Density

Corneal endothelial cell density (ECD) was evaluated as a safety parameter in this study. Paired corneal endothelial cell density measurements were available for six eyes. The mean preoperative ECD was 1822.3 ± 537.7 cells/mm^2^, and the mean postoperative ECD at the final follow-up visit was 1636.7 ± 741.1 cells/mm^2^. The difference was not statistically significant (*p* = 0.287, paired *t*-test). For four additional eyes, only postoperative values were available due to preoperative corneal opacity. Specular microscopy was not performed in the remaining eye.

**Figure 2:**
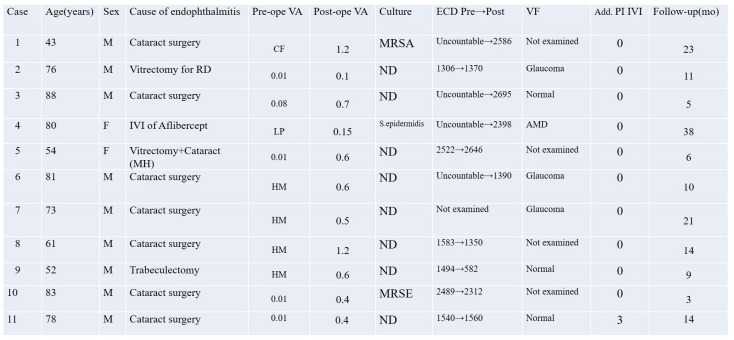
Clinical summary of 11 eyes with infectious endophthalmitis treated using intravitreal 1.25% PI and low-dose antibiotic irrigation. The table includes patient demographics, etiology, pre- and postoperative visual acuity, corneal endothelial cell density, Goldmann visual field findings, culture results, and additional intravitreal injections of PI. MH = macular hole; AMD = age-related macular degeneration; ECD = endothelial cell density; IVI = intravitreal injection; PI = povidone-iodine.

These results suggest that the treatment protocol did not result in clinically significant corneal endothelial damage. The individual values are summarized in Figure 2.

### 3.3. Visual Field Findings

Goldmann perimetry was performed for 7 out of the 11 eyes as part of postoperative visual function assessment. Among these, four eyes exhibited visual field defects attributable to pre-existing conditions: three cases of glaucoma and one of age-related macular degeneration (AMD). The remaining three eyes showed normal or functionally preserved visual fields. No apparent postoperative visual field defects were observed that could be attributed to the treatment protocol. The visual field results are summarized in Figure 2.

### 3.4. Microbiological Findings

**Figure 3:**
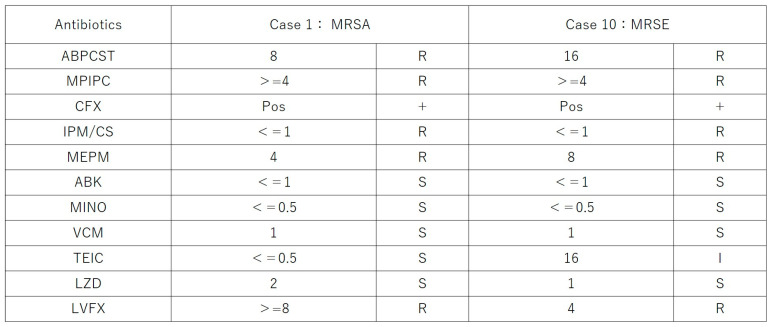
Drug susceptibility profiles of multidrug-resistant isolates from two cases of infectious endophthalmitis. Case 1: methicillin-resistant *Staphylococcus aureus* (MRSA); Case 10: methicillin-resistant *Staphylococcus epidermidis* (MRSE). Susceptibility testing was performed for a range of antibiotics using standard methods. R = resistant; S = susceptible; I = intermediate. Pos = positive result indicating methicillin resistance (screening using cephalexin [CFX]). ABPCST = ampicillin/sulbactam; MPIPC = mezlocillin; CFX = cefalexin; IPM/CS = imipenem/cilastatin; MEPM = meropenem; ABK = arbekacin; MINO = minocycline; VCM = vancomycin; TEIC = teicoplanin; LZD = linezolid; LVFX = levofloxacin.

Microbiological cultures were performed in all 11 cases using vitreous and/or aqueous samples obtained at multiple time points, including before intravitreal PI injection, before the start of vitrectomy, and at the conclusion of surgery. Positive culture results were identified in three cases (27.3%): methicillin-resistant *Staphylococcus aureus* (MRSA) in Case 1, methicillin-resistant *Staphylococcus epidermidis* (MRSE) in Case 10, and *Staphylococcus epidermidis* in Case 4. All other cases yielded negative culture results despite clinical features consistent with infectious endophthalmitis. No fungal or polymicrobial infections were detected in this series. These results are summarized in Figure 2. The antibiotic resistance profiles of the MRSA and MRSE isolates are shown in Figure 3.

### 3.5. Adverse Events

No adverse events such as retinal toxicity, optic nerve injury, or intraocular pressure elevation were observed in any of the 11 cases. A repeated intravitreal injection of 1.25% PI was administered postoperatively in one case (Case 11), and it was well tolerated without any signs of ocular toxicity. There were no instances of recurrent infection, persistent intraocular inflammation, or treatment-related complications.

These findings support the ocular safety of the PI-based combination treatment protocol.

### 3.6. Overall Clinical Outcome and Representative Case

The follow-up period ranged from 3 to 38 months, with a mean of 14.6 ± 9.7 months and a median of 11 months. “Final follow-up” refers to the latest clinical evaluation during this follow-up period. All 11 eyes achieved clinical resolution of infectious endophthalmitis following the combination treatment protocol, with no recurrence, persistent inflammation, or severe complications observed during follow-up.

Case 11 was a particularly challenging case of postoperative endophthalmitis following cataract surgery. The patient had previously undergone two pars plana vitrectomies and intraocular lens explantation at another institution, but inflammation recurred postoperatively. Our treatment protocol was initiated upon referral, and three additional intravitreal injections of 1.25% PI were administered postoperatively to control residual intraocular inflammation. Complete resolution was achieved without adverse effects.

**Figure 4:**
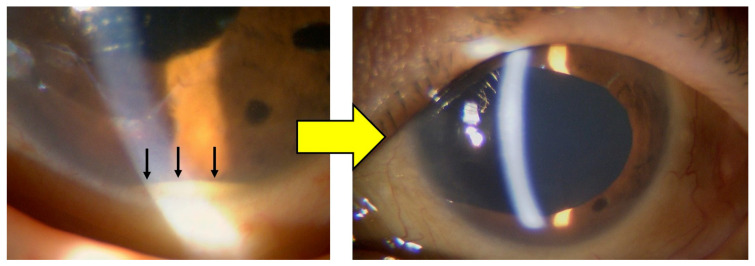
Clinical course of Case 11, which required additional intravitreal injections of 1.25% PI. The left image shows anterior chamber hypopyon (arrow), indicating active intraocular inflammation at presentation. The right image demonstrates the resolution of endophthalmitis following treatment. The eye is aphakic due to prior intraocular lens explantation performed at another facility following two unsuccessful vitrectomies for post-cataract endophthalmitis. Complete resolution of inflammation was achieved without adverse events, and visual acuity improved from 0.01 to 0.4.

Representative clinical images from Case 11 are presented in Figure 4, showing the resolution of intraocular inflammation and visual recovery.

## 4. Discussion

The present study demonstrates that a combination of intravitreal injection of 1.25% PI and vitrectomy using low-dose antibiotic-enriched irrigation can effectively resolve infectious endophthalmitis. All 11 eyes achieved clinical resolution without recurrence or any vision-threatening complications. This result supports the potential of PI-based disinfection as a safe and broad-spectrum alternative or adjunct to conventional intravitreal antibiotics. Notably, although the proposed protocol included preoperative intravitreal PI injection, this step was omitted in five patients (Cases 2–6) due to time constraints or direct referral for urgent surgery. Despite this deviation, all eyes achieved infection resolution, and visual outcomes remained favorable, suggesting that the overall efficacy of the protocol was maintained.

Our findings build upon previous investigations, including our own prior reports of using 0.025% PI in an irrigation solution during vitrectomy for endophthalmitis [18] and combining it with intravitreal PI injection. In the present study, we selected a 1.25% PI concentration for intravitreal injection, based on our previously established protocol in which this dose, when diluted in an assumed vitreous volume of 5 mL, yields an intraocular concentration of 0.025%—the same concentration shown to be safe and effective in our earlier irrigation studies [17,18]. The current protocol is unique in that PI was excluded from the irrigation fluid, and instead, low concentrations of vancomycin (20 µg/mL) and ceftazidime (40 µg/mL) were added to reduce the risk of HORV while maintaining antimicrobial synergy. Notably, recent in vitro studies have demonstrated that the combination of PI and vancomycin exhibits synergistic effects against immature biofilms, supporting the rationale for their combined use while minimizing individual toxicity risks [19]. The antibiotic concentrations used in this study—vancomycin at 20 µg/mL and ceftazidime at 40 µg/mL—were selected based on the Japanese national emergency protocol for infectious endophthalmitis published in 2005 by Usui et al., which remains widely utilized in clinical practice in Japan [20]. In the original protocol, intravitreal injections of vancomycin (1 mg) and ceftazidime (2 mg) were administered. In our modified approach, we replaced these bolus injections with intravitreal PI and used low-dose antibiotics only in the irrigation solution, which still achieved concentrations exceeding the minimum inhibitory concentrations (MICs) for major pathogens, as vancomycin typically demonstrates MICs of ≤2 µg/mL for Gram-positive organisms such as MRSA and MRSE, while ceftazidime shows MICs of ≤8 µg/mL for Gram-negative organisms such as *Pseudomonas aeruginosa* and *E. coli* [21]. This strategy aims to maintain antimicrobial coverage while minimizing the potential for drug-related complications, including hemorrhagic occlusive retinal vasculitis (HORV), and leverages the broad-spectrum and synergistic activity of PI to enhance therapeutic efficacy with reduced toxicity risk. Furthermore, all cases received intravitreal PI injection at the conclusion of surgery, and one case (Case 11) received additional postoperative PI injections on days 2, 4, and 6 due to persistent inflammation, all without evidence of ocular toxicity.

Unlike previous protocols that included PI in the irrigation solution, this study employed a modified approach by omitting PI from the infusion fluid altogether. Although our earlier studies demonstrated the efficacy of 0.025% PI irrigation [17,18], it has become clear that PI loses its antimicrobial activity within approximately 15 min when diluted in BSS PLUS, necessitating the replacement of the irrigation bottle once during the procedure—typically between anterior chamber and vitreous cavity lavage—to maintain antimicrobial efficacy. This rapid loss of activity is likely due to weak reducing agents present in BSS PLUS—such as oxidized glutathione, calcium chloride dehydrate, magnesium chloride hexahydrate, and dextrose—which may promote the conversion of free iodine (povidone/nHI_3_, I_3_^−^, and I_2_) to iodide ion (I^−^), which lacks bactericidal activity [17]. While this protocol is feasible and has been used successfully, it adds procedural complexity and raises concerns regarding corneal endothelial safety, particularly in eyes with prior ocular surgery or inflammation. PI is known to cause direct cellular membrane disruption, and continuous irrigation over the corneal endothelium may pose a risk of toxicity.

Although our previous studies did not reveal measurable damage to endothelial cells [17,18], concerns remain that continuous irrigation with povidone-iodine, especially in eyes with pre-existing corneal endothelial compromise, may pose a risk of toxicity due to its direct membrane-disruptive effects. Therefore, in the present study, we adopted a safer and simpler approach by focusing on intravitreal injection of 1.25% PI as the main disinfection strategy.

PI has several advantages over traditional antibiotics, including its rapid bactericidal action [22], efficacy against bacteria [11], fungi, viruses [13], and biofilms [14], and the absence of microbial resistance [15]. In our study, two multidrug-resistant organisms (MRSA and MRSE) were successfully treated with this protocol, further supporting the broad utility of PI in modern antimicrobial ophthalmic practice. This finding is consistent with prior reports highlighting the challenges posed by antibiotic-resistant pathogens in endophthalmitis and the potential role of antiseptics such as PI in overcoming these limitations [23,24]. Notably, no adverse events related to PI toxicity were detected based on visual field testing or corneal endothelial cell measurements. The visual outcomes in our series were also favorable, with a mean final BCVA of 0.296 logMAR. Although the direct influence of PI on visual recovery remains uncertain, our results demonstrated a favorable visual outcome. Only 1 out of 11 eyes (9.1%) had a final visual acuity (VA) worse than 0.1, even in the presence of multidrug-resistant organisms such as MRSA and MRSE. In comparison, a recent multicenter cohort study conducted in Japan by Yoshimura et al. reported that 23.1% of eyes developed legal blindness at 12 weeks after treatment initiation [1]. While differences in treatment strategies and patient backgrounds must be considered, our findings suggest that PI-based therapy may contribute to better visual preservation in infectious endophthalmitis, even in severe or resistant cases.

The effectiveness and safety of intraoperative PI-containing irrigation have also been supported by independent studies. Mi et al. reported favorable outcomes in a series of 12 eyes with endophthalmitis, achieving clinical and microbiological improvement in 11 eyes and visual acuity improvement in 10 eyes, without signs of intraocular toxicity [25]. Similarly, Sakata et al. described two cases of bleb-related endophthalmitis successfully treated using PI irrigation, with not only clinical resolution but also functional recovery evidenced by improvements in both a-wave and b-wave amplitudes on skin electrode ERG [26]. These reports reinforce the reproducibility and therapeutic potential of PI-based intraoperative disinfection strategies.

In addition, we have previously reported a case of endogenous endophthalmitis successfully treated with intravitreal injection of 1.25% PI without vitrectomy or intravitreal antibiotics [27]. This suggests that PI monotherapy may have potential as a first-line option in early-stage or less severe cases, particularly when surgical intervention is not feasible.

Furthermore, PI has been reported to exhibit not only antimicrobial activity but also antioxidant [28] and anti-inflammatory activity [29]. These pharmacological properties may contribute to the favorable functional outcomes observed in our study.

PI is widely available and inexpensive, making it a practical option globally, especially in resource-limited settings. Although PI is commercially prepared as a sterile solution, it is not a terminally sterilized pharmaceutical product. To enhance sterility and minimize the risk of contamination, we filtered the diluted 1.25% PI solution through a 0.22 µm membrane filter prior to intravitreal injection. This additional step was taken in light of past reports of PI-associated infectious outbreaks and underscores the importance of aseptic precautions when preparing intravitreal formulations. One such outbreak involved intrinsic contamination of a povidone-iodine product with *Pseudomonas cepacia*, leading to multiple infections and pseudoinfections—including peritonitis and pseudobacteremia—across healthcare facilities in the United States. Isolates from patients and PI bottles showed identical resistance patterns and genetic profiles, confirming the link to a single contaminated lot [30]. This enhances its feasibility for implementation in low-resource environments. These characteristics highlight the potential utility of PI-based therapies not only in advanced medical centers but also in developing countries where access to antibiotics or surgical expertise may be limited.

Limitations of this study include its retrospective design, small sample size, and lack of a control group. A formal sample size calculation was not performed, as this was a retrospective study of all consecutive cases treated with PI-based therapy at our institution. Nevertheless, the small sample size limits statistical power and generalizability, and the results should be interpreted with caution. Importantly, all 11 cases achieved clinical resolution of infection, which provides compelling preliminary support for the efficacy of this approach despite the limited sample size. In addition, the relatively low culture positivity rate (27.3%) may reflect diagnostic limitations, including the use of 27-gauge trans-pars plana aspiration (TAP) under poor visualization conditions due to media opacity. Although aqueous and vitreous samples were collected prior to the initial intravitreal PI injection, diagnostic yield may have been limited. Furthermore, two patients had received antibiotic treatment at referring institutions before presentation—one received intravitreal, anterior chamber, and subconjunctival injections, and the other received a subconjunctival injection—which may have reduced microbial viability. Although it would have been ideal to include a control group receiving standard therapy (i.e., intravitreal antibiotics alone), this was not feasible due to ethical concerns, as PI-based therapy has become the standard of care at our institution following previously published favorable outcomes. The absence of a contemporaneous control group limits direct comparison and weakens causal inference regarding the efficacy of the treatment. Notably, due to ethical considerations, creating a control arm without the use of PI was not feasible in our institution, as PI-based therapy has become our standard of care following previously published favorable outcomes. All patients strongly preferred to receive PI-based treatment after being informed of prior results. Although we did not perform a statistical comparison with previous methods in this study, indirect reference to the visual outcomes reported by Yoshimura et al. [1] suggests a potentially better result in our series. However, such comparisons are inherently limited due to differences in patient backgrounds and treatment settings. Future prospective studies with larger sample sizes, ideally incorporating matched or randomized designs, are warranted to confirm the benefits and clarify the optimal role of PI-based therapy in the management of infectious endophthalmitis.

## 5. Conclusions

This study demonstrates that the combination of intravitreal 1.25% PI injection and vitrectomy with low-dose antibiotic irrigation is a feasible and effective approach for treating infectious endophthalmitis. The protocol showed a favorable safety profile and promising visual outcomes, even in the presence of multidrug-resistant pathogens. The broad-spectrum efficacy, accessibility, low cost, and simple preparation of PI make it a compelling option for clinical use, particularly in resource-limited settings. Further validation through controlled studies may support wider adoption of this protocol and investigation into PI monotherapy for selected cases.

## Data Availability

The data presented in this study are available upon request from the corresponding author. The data are not publicly available due to privacy and ethical restrictions.

## Supplementary Materials

The following supporting information can be downloaded at: https://www.mdpi.com/article/10.3390/pharmaceutics17080995/s1, Figure S1: Flowchart summarizing the treatment protocol used in this study, alongside previously reported protocols for comparison [17,18]; Figure S2: Preparation set for the 1.25% povidone-iodine solution used for intravitreal injection, including povidone-iodine 10%, sterile normal saline, syringes, needles, and a 0.22 μm membrane filter; Figure S3: Step-by-step photographs showing the preparation procedure for the 1.25% povidone-iodine solution. Panels A–F indicate the sequential steps of the preparation process.

## Abbreviations

The following abbreviations are used in this manuscript:

PI	Povidone-Iodine
IVI	Intravitreal Injection
BCVA	Best-Corrected Visual Acuity
ERG	Electroretinography
BSS	Balanced Salt Solution
MRSA	Methicillin-Resistant *Staphylococcus aureus*
MRSE	Methicillin-Resistant *Staphylococcus epidermidis*
SE	*Staphylococcus epidermidis*
HORV	Hemorrhagic Occlusive Retinal Vasculitis
IRB	Institutional Review Board
SD	Standard Deviation
logMAR	Logarithm of the Minimum Angle of Resolution
RCT	Randomized Controlled Trial
