# Supplementary figures and images for: Intravitreal Povidone-Iodine Injection and Low-Dose Antibiotic Irrigation for Infectious Endophthalmitis: A Retrospective Case Series
Source: Pharmaceutics. 2025 Jul 31;17(8):995. doi: 10.3390/pharmaceutics17080995 (PMC12389214; doi:10.3390/pharmaceutics17080995)

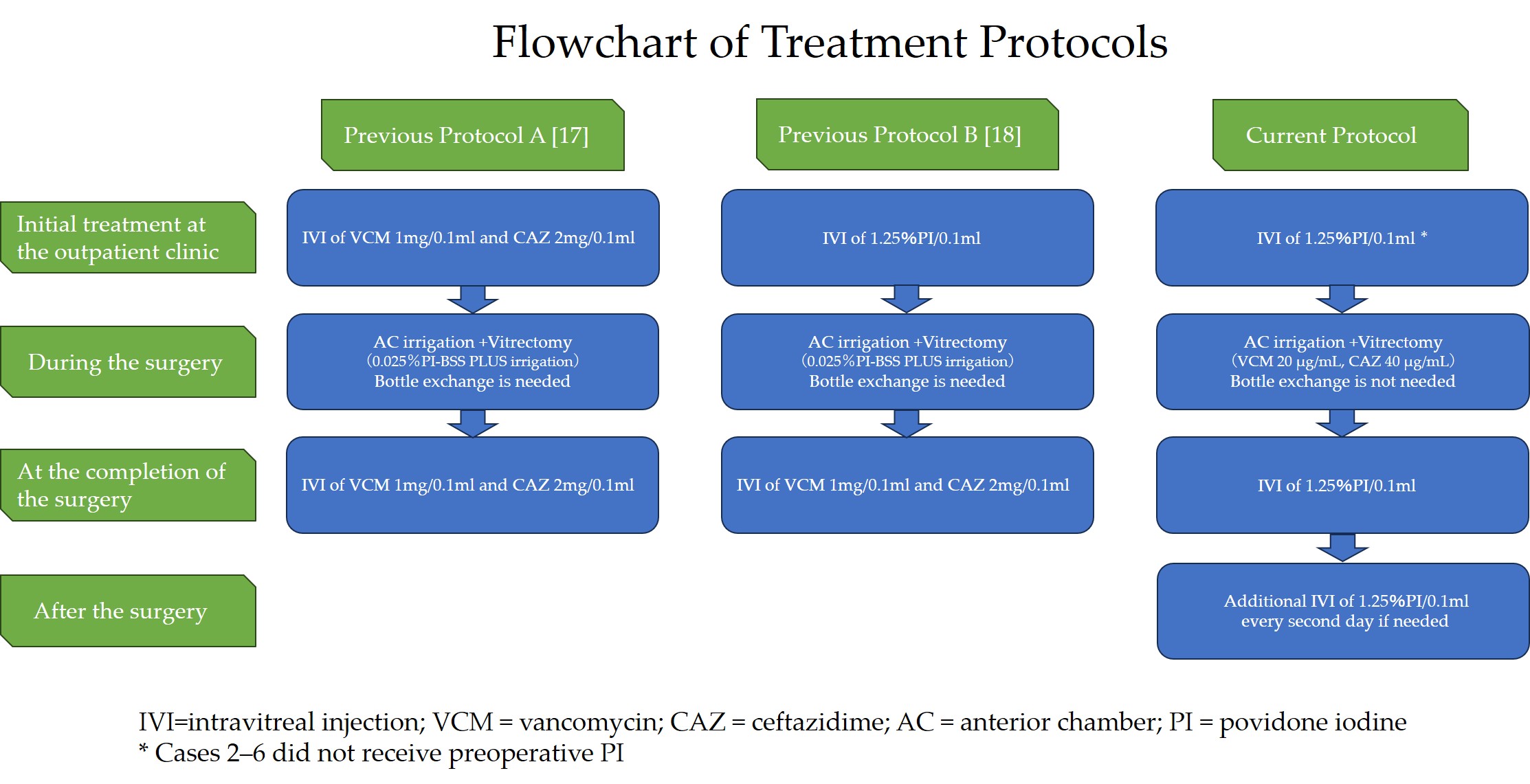

Supplement: Supplementary file 1 [file pharmaceutics-17-00995-s001.zip › Supplementary Figure S1 Protocol .jpg]

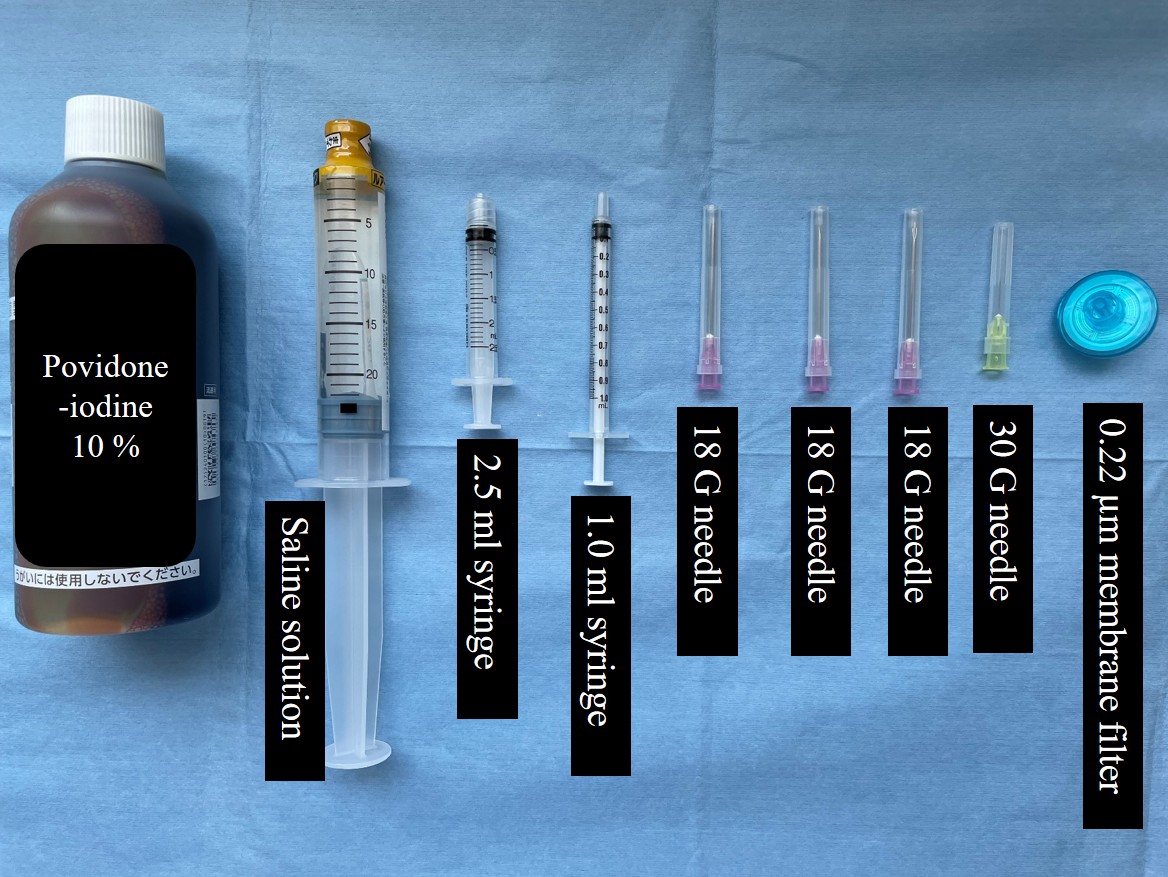

Supplement: Supplementary file 1 [file pharmaceutics-17-00995-s001.zip › Supplementary Figure S2.jpg]

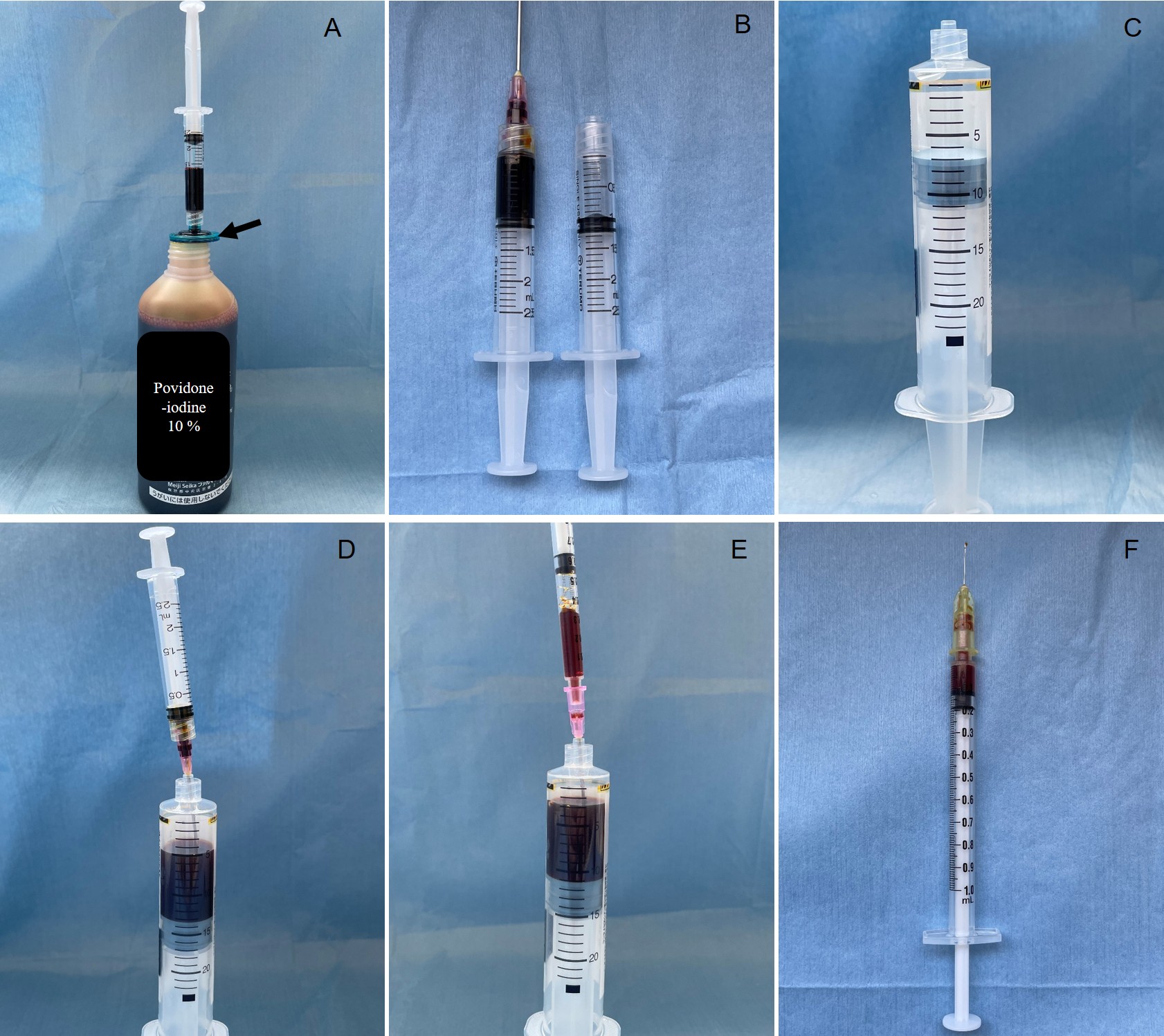

Supplement: Supplementary file 1 [file pharmaceutics-17-00995-s001.zip › Supplementary Figure S3.jpg]
